# Perceptual space and adjective rating of 2.5D tactile patterns

**DOI:** 10.1038/s41598-025-88334-4

**Published:** 2025-02-04

**Authors:** Inwook Hwang, Sungryul Yun, Jaeyoung Park

**Affiliations:** 1 Tangible Interface Creative Research Section, Electronics and Telecommunications Research Institute (ETRI), Daejeon, 34129 Korea; 2 Department of Computer Engineering, Hongik University, Seoul, 04066 Korea

**Keywords:** Computer science, Psychology

## Abstract

The present study investigates the human haptic perception of 2.5D tactile patterns based on adjective ratings and how physical factors, such as the bump diameter of the pattern or material, affect their tactile perception. We designed fifty tactile patterns by varying the pattern’s bump diameter, pattern uniformity, and material and evaluated the effect of the parameters on haptic perception by conducting a couple of human subject experiments. In Experiment 1, the perceived intensities of the tactile patterns were tested for a total of ten properties (adjective pairs). The experimental results indicate significant effects of the factors, the bump diameter, pattern type, and material on the perceived intensities of the 2.5D patterns. In Experiment 2, a cluster sorting of the tactile patterns was conducted, and a haptic perceptual space was constructed with an MDS (multi-dimensional scaling). The results indicate a grouping of the samples by bump diameter and an effect of sample pattern uniformity for larger 2.5D tactile patterns. Overall, the present study showed that bump diameter, pattern type, and material significantly affected the perception of 2.5D tactile patterns based on the adjective ratings, and the 2.5D patterns could be grouped by the pattern’s bump diameter and uniformity.

## Introduction

From its early stage, a smart device has a specific form factor, having an input interface as a touch screen where a user can input information with bare fingers. While the touch screen has evolved to feature a high-resolution visual display, the user can feel the sensation of touch only through vibration. On the other hand, a surface-morphing tactile display, a more recent type of 2.5D display that can render the surface of a touch screen with embossed bumpy patterns like a relief^1–9^. This type of tactile display can convey information such as symbols and images, as well as qualitative expressions based on tactile sensations to trigger intended emotions in users. The 2.5D displays include the older forms of haptic display, such as a refreshable braille display, belong to the category^10^. However, when we implement a 2.5D display on a smart device such as a tablet, we still need to know how to configure the display parameters, including the display material, tactile resolution, and pattern regularity. For this reason, we were prompted to evaluate how humans perceive 2.5D tactile patterns and analyze the effect of parameters on the human perception of tactile patterns.

Multiple studies in neurophysiology have been conducted to see how and what type of signals are generated and processed surface features by stroking surface features, like 2.5D surface features. An object’s tactile property is sensed via four different kinds of mechanoreceptors^11,12^, of which Pacinian corpuscles and Merkel disks react to the surface’s bumpiness or roughness^13^. Especially, low-level cutaneous perception of a 2.5D (planar array with height) pattern can be accounted by the responses of slowly adapting type 1 (SA1) and rapidly adapting (RA) mechanoreceptors^14^. In particular, SA1 afferents are found to have characteristics that are closely related to the perception of 2.5D tactile patterns. Johnson et al. demonstrated that the SA1 afferents respond actively to surface features like edges and cylindrical shapes^15,16^. They are known to respond linearly to skin indentation. In addition, the roles of SA1 and RA mechanoreceptors as the fingertip touches curved surface shapes or surfaces with raised dots were identified^13,17,18^. The aforementioned findings can exclusively explicate how tactile stimuli stimulate low-level mechanoreceptors. However, understanding of higher-level perception of 2.5D tactile patterns in terms of verbal adjectives still requires further investigation.

Previous studies in the field of psychophysics and haptics indicate the significance of surface feature in the interaction with smart devices^19^. A substantial amount of studies on human perception of surface features such as bumpiness or gratings show that they can significantly affect the interaction with the environment. For example, Kitada et al.’s study showed the increase of unpleasantness as a power function of roughness^20^. It was also found that the tactile information enhanced the interaction with the touchscreen^21^. However, there is still a need for the data on how humans perceive 2.5D patterns based on a wide range of adjective intensities and perceptual space for an effective rendering of 2.5D tactile patterns. For example, if we want to create the sensation of sharpness at a certain level with a 2.5D tactile pattern on a touchscreen, there is yet to be an extensive reference.

Human perception of haptic stimuli can be analyzed in various aspects, e.g., precision or accuracy that can be estimated with psychophysical experiments. Among them, the perceptual space and adjective ratings provide high-level information on how human perceives haptic stimuli. The perceptual space is an n-dimensional mathematical space that can visually represent the relationship between the percepts of haptic stimuli^22^. A typical method that helps reveal the underlying structure of the perceptual space is using Multi-Dimensional Scaling (MDS)^23^. In the field of haptics, many researches have been conducted to investigate the perceptual dimension of vibrotactile stimuli^24–28^.

The present study aims to achieve knowledge of how humans perceive 2.5D tactile patterns based on adjective ratings and how physical factors, such as the bump diameter of the pattern or material, affect their tactile perception. Moreover, this study analyzes how the 2.5D tactile patterns are distributed in the perceptual space formed by the adjective pairs using the MDS. Eventually, the tactile patterns perceived in a similar manner are grouped together, which will benefit the optimal rendering of the 2.5D tactile patterns by a surface-morphing tactile display. Then, the major contributions of this study are summarized as (i) quantitatively analyzing the effect of parameters on 2.5D tactile pattern perception and (ii) reporting the distribution and grouping of 2.5D tactile patterns for a perceptual space formed by multiple adjective pairs to describe the 2.5D patterns. To achieve the goal, we conducted two experiments with fifty 2.5D tactile pattern samples whose bump diameter (spatial density), pattern uniformity, and materials varied. In the first experiment, the adjective ratings of the 2.5D patterns for different parameters, including the pattern’s material and bump diameter, were collected, and their effect was analyzed. In the next experiment, we estimated the pairwise dissimilarities of the samples by a perceptual experiment using the cluster sorting procedure^29^. Then, an Euclidean perceptual space is formed based on adjective pairs, and the distribution and grouping of the 2.5D tactile patterns are derived. The findings from this study will serve as haptic rendering specifications for a surface-morphing tactile display regarding the decision of the display’s surface material, resolution of patterns, and the types of tactile pattern groups.

## Experiment 1: adjective rating of 2.5D tactile patterns

**Fig. 1:**
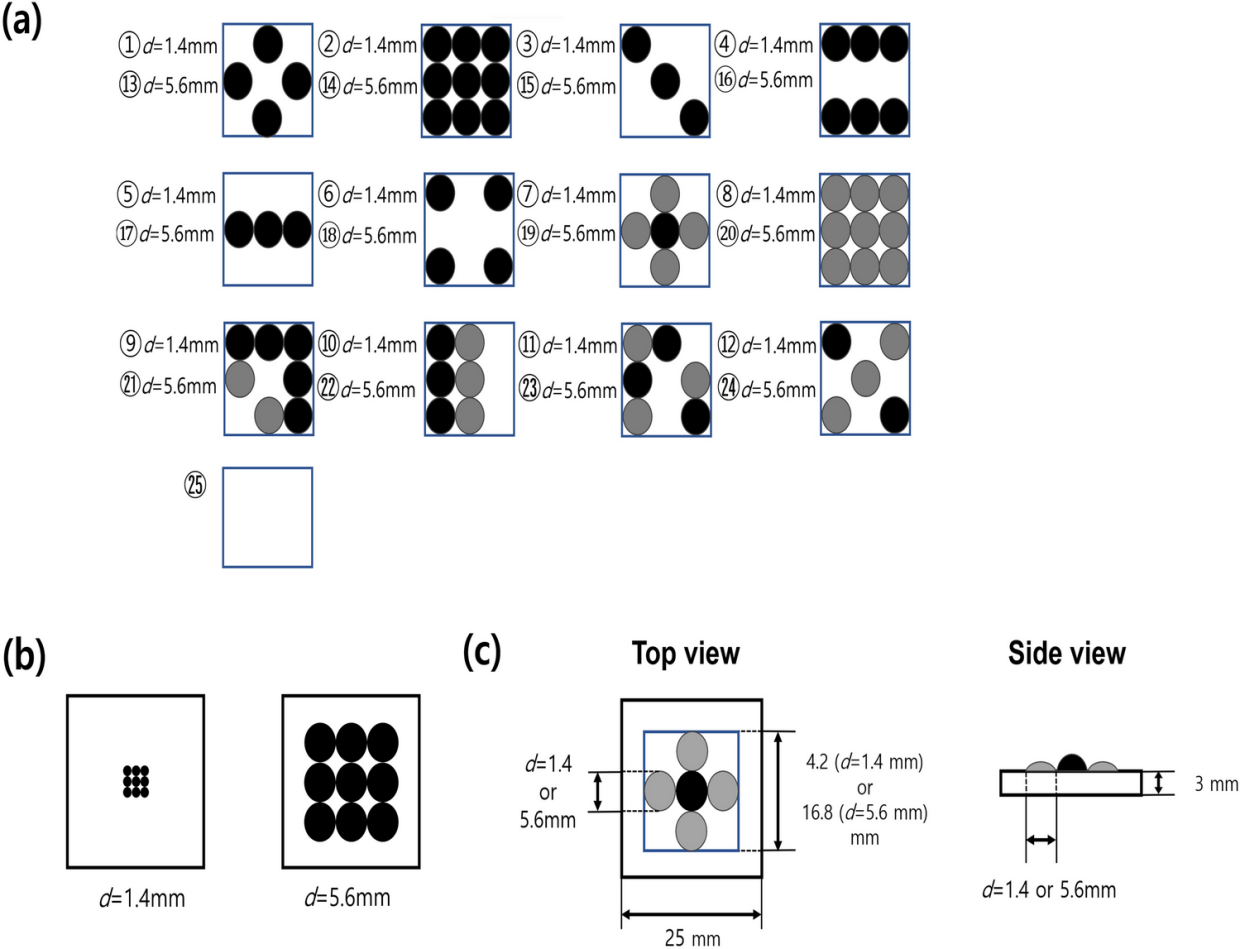
2.5D tactile pattern designs for the experiment. (**a**) Twenty five 2.5D tactile patterns for the experiments. On each square cell of a sample were located multiple dome shapes, i.e., bumps. For each pattern, samples with bumps having fixed diameter values (*d*  =  1.4 and 5.6 mm) were fabricated. Three colors (black, gray, and white) mean the height difference in the dome height. (**b**) Size comparison of samples with *d*  =  1.4 and 5.6 mm (**c**) Top and side views of a 2.5D pattern sample. Each pattern is embedded on a flat square plate with a side length of 25 mm.

In this experiment, the adjective intensity of the 2.5D tactile patterns was rated for fifty patterns with varied pattern properties (Fig. 1a). The purpose of the experiment is to see how the descriptive intensity of the 2.5D tactile pattern is distributed in the perceptual space, and how physical factors, including the material, bump diameter of the pattern, and the pattern’s uniformity.

The experimental methods were approved by Hongik University Institutional Board with No. 7002340-202203-HR-002 and the experiment was performed in accordance with the Declaration of Helsinki.

### Methods

#### Participants

Twenty-four participants (eleven males and thirteen females; 18–26 years old, mean±SD: 21.6±2.2 yrs) were recruited for the experiment. The participants had no known sensory disorder by self-reporting and were compensated after the experiment.

The experiment was conducted twice for each participant. Informed consent was obtained from all participants.

#### Stimuli

**Table 1:** Dome height by the diameter *d*.

Color	*d* = 1.4 mm	*d* = 5.6 mm
Black	$360~\upmu \!{\hbox {m}}$	$1440~\upmu \!{\hbox {m}}$
Gray	$120~\upmu \!{\hbox {m}}$	$480~\upmu \!{\hbox {m}}$
White	0 (flat)	

Twelve 2.5D tactile patterns were designed as shown in Fig. 1a. The sample patterns were designed to be representative of the possible combinations in a 3$\times$3 array, considering the symmetry, the variance of the height, and the experimental procedure. Multiple dome shapes, i.e., bumps in two diameters, 1.4 and 5.6 mm, were located on each square cell of a sample (Fig. 1b). The diameter of the smaller dome shape, 1.4 mm, was the target resolution of the surface-morphing tactile display that we developed in a previous study^1^. The diameter of the larger dome sample, 5.6 mm, was decided to be four times that of the smaller ones to ensure a distinct perceptual difference between the samples in different sizes. Each dome shape had two heights as shown in Table 1. To avoid the error in fabricating the sample, we decided to make the height of the dome shape a multiple of the height resolution. A black circle in Fig. 1c indicates a taller bump, while a grey circle means a shorter one. The patterns 1–6 (13–18) were designed to be symmetric and have only taller bumps. Patterns 8 and 20 were designed to have only shorter bumps. Meanwhile, we designed the patterns 7, 9–12 (19, 21–24) to have bumps of two different heights to see the effect of height variation of a pattern. Each sample was 3D printed (Stratasys Objet 300, height resolution of 28 $\upmu \!{\hbox {m}}$), being embedded on a square-shaped flat surface whose size is described in Fig. 1c. Including a flat surface, a total of twenty-five (1 flat + 2 diameters$\times$12 patterns) 2.5D tactile patterns were designed. The samples were fabricated in two different materials with the softness of 0.5–1.0 MPa (*soft*, TangoPlus by Stratasys) and 2.2–3.0 GPa (*hard*, Vero Black by Stratasys). Including the flat surface, a total of fifty (25 $\times$ 2(soft/hard)) 2.5D tactile pattern samples were fabricated.

#### Apparatus

**Fig. 2:**
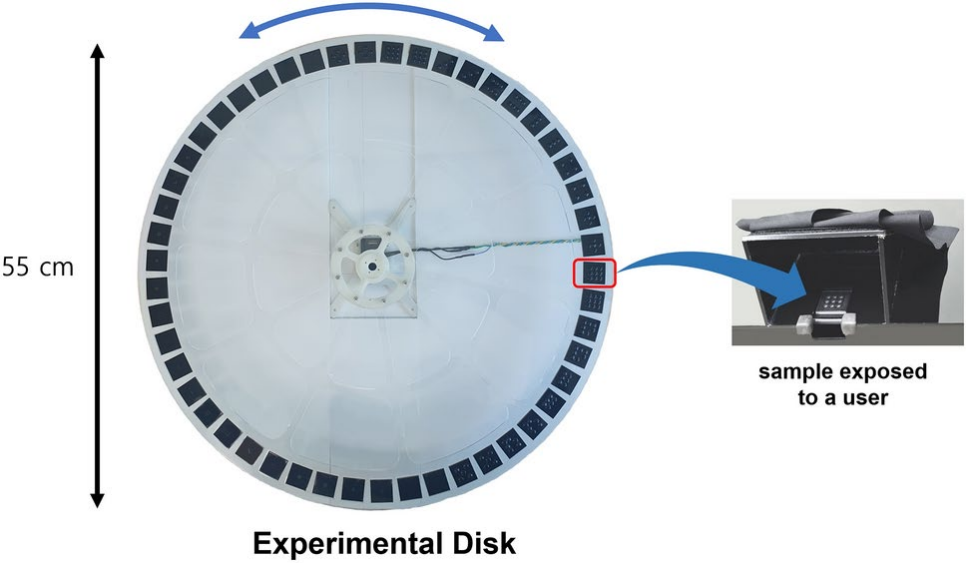
A disk for Experiment 1 with 50 samples. Only one sample is exposed outside, and a black cloth is installed at the opening, avoiding a possible visual cue during the experiment.

For this experiment, we built an apparatus that can present a 2.5D tactile pattern to a participant in a random order, as requested by a computer program made in Visual Studio C++, using OpenGL (Fig. 2). The experimental apparatus contains a rotating disk mechanism inside a box blocking the visual cue of the samples to the participant. We made a disk with a diameter of 55 cm, where all fifty samples described in the previous section were installed. As shown in Fig. 2, when the rotating disk is installed inside the box, only one sample is exposed outside. A support structure is installed beneath the opening to ensure a stable touch with samples. The opening was covered with a black cloth to avoid possible visual cues on the samples. A computer program can control what sample to be exposed with a Dynamixel smart actuator (XC430-W15Q-T, Robotis, Inc., Korea) whose angular position can be controlled by a microcontroller (Arduino Uno, Italy). The command to move the desired sample to the opening from the computer program to the microprocessor is sent via serial communication.

#### Procedure

**Fig. 3:**
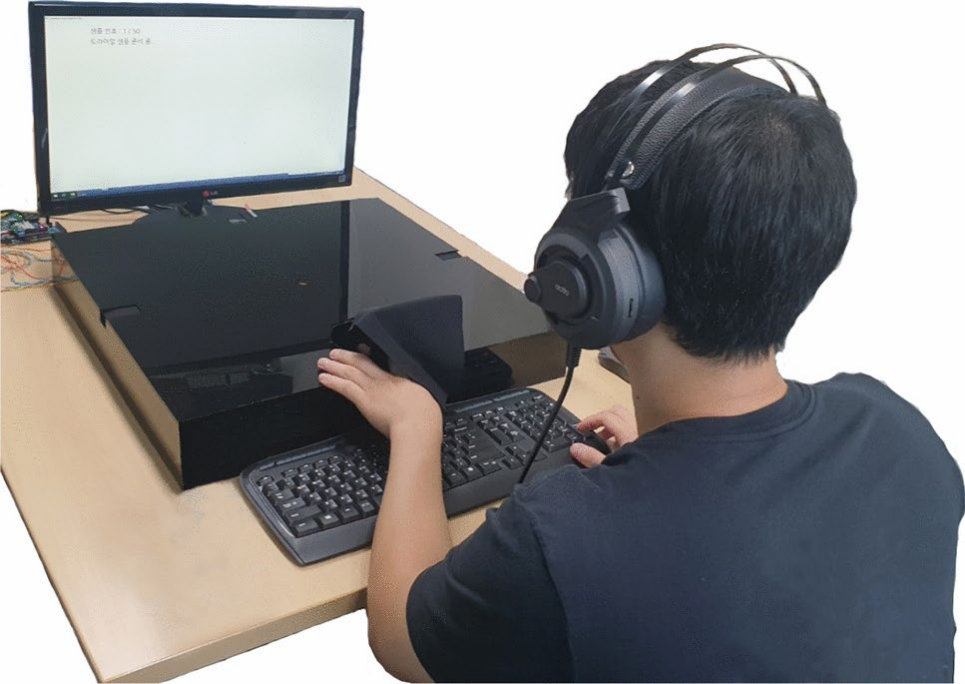
Setup for experiment 1.

**Table 2:** Adjective pairs used for Experiment 1 (Translated from Korean).

Pair #	Property	Adjective 1	Adjective 2
1	Stiffness	Hard	Soft
2	Micro-roughness	Rough	Smooth
3	Macro-roughness	Uneven	Flat
4	Spatial regularity	Aligned	Jagged
5	Viscoelasticity	Sticky	Slippery
6	Sharpness	Sharp	Blunt
7	Spatial density	Dense	Sparse
8	Weight	Light	Heavy
9	Clarity	Clear	Vague
10	Brightness	Bright	Dark

They were selected from previous studies on human perception of real textures felt by bare hands^23,30–32^.

Before the experiment, a participant signed a consent form and was instructed about the experimental procedure. Then, the participant was seated in front of an experimental computer and wore headphones where white noise was played during the experiment to block possible noise from the experimental apparatus (Fig. 3). At the beginning of the experiment, the participant typed their ID with the keyboard. When the experimental program launched, the participant entered his/her name using the keyboard. Then, a training session was provided by the experimental program, which had the layout the same as the main experiment except that only one sample was randomly picked for the test. On the screen, ten adjective pairs were present with slide bars, each of which had an adjective on each end with a range between 0 and 200. Each pair consisted of two adjectives with contrary meanings. Table 2 shows the ten adjective pairs used for the experiment, which were selected from perceptual dimensions for bare-finger perception of real textures^23,30–32^. From the haptic properties listed in the previous studies, we excluded the warmness (warm/cold) since it could be implemented with a specific type of thermal haptic display, which we judged to be out of the current study’s scope. There were seven pairs directly representing haptic properties (pairs #1 to #8: stiffness, roughness (micro and macro), spatial regularity, viscoelasticity, sharpness, and weight) and two pairs related to visual properties (pairs #9 to #10: clarity and brightness).

All slide bars were positioned in the middle, corresponding to the value of 100. The participant felt the random training sample with the major hand. Then s/he moved the slide bar to indicate how they felt about the sample. Once ready, the participant could move to the main experiment by hitting the enter key. Then, a randomized order of a total of fifty samples was created, and the samples were presented to the participants. On each trial, a sample was moved to the opening of the experimental apparatus as predetermined so that the participant could touch and feel it. White noise was played on the headphones while the sample was moving to the destination to block possible audio cues from the experimental apparatus. Then, they rated it by moving the slide bars as was for the training session.

#### Data analysis

The effects of three factors, material, bump diameter, and the pattern on the adjective rating, were analyzed for the ten properties in Table 2 with a three-way ANOVA. For each property, i.e., an adjective pair, a Tukey’s HSD multiple comparison test was conducted to find statistically distinguishable 2.5D sample groups.

### Results

**Fig. 4:**
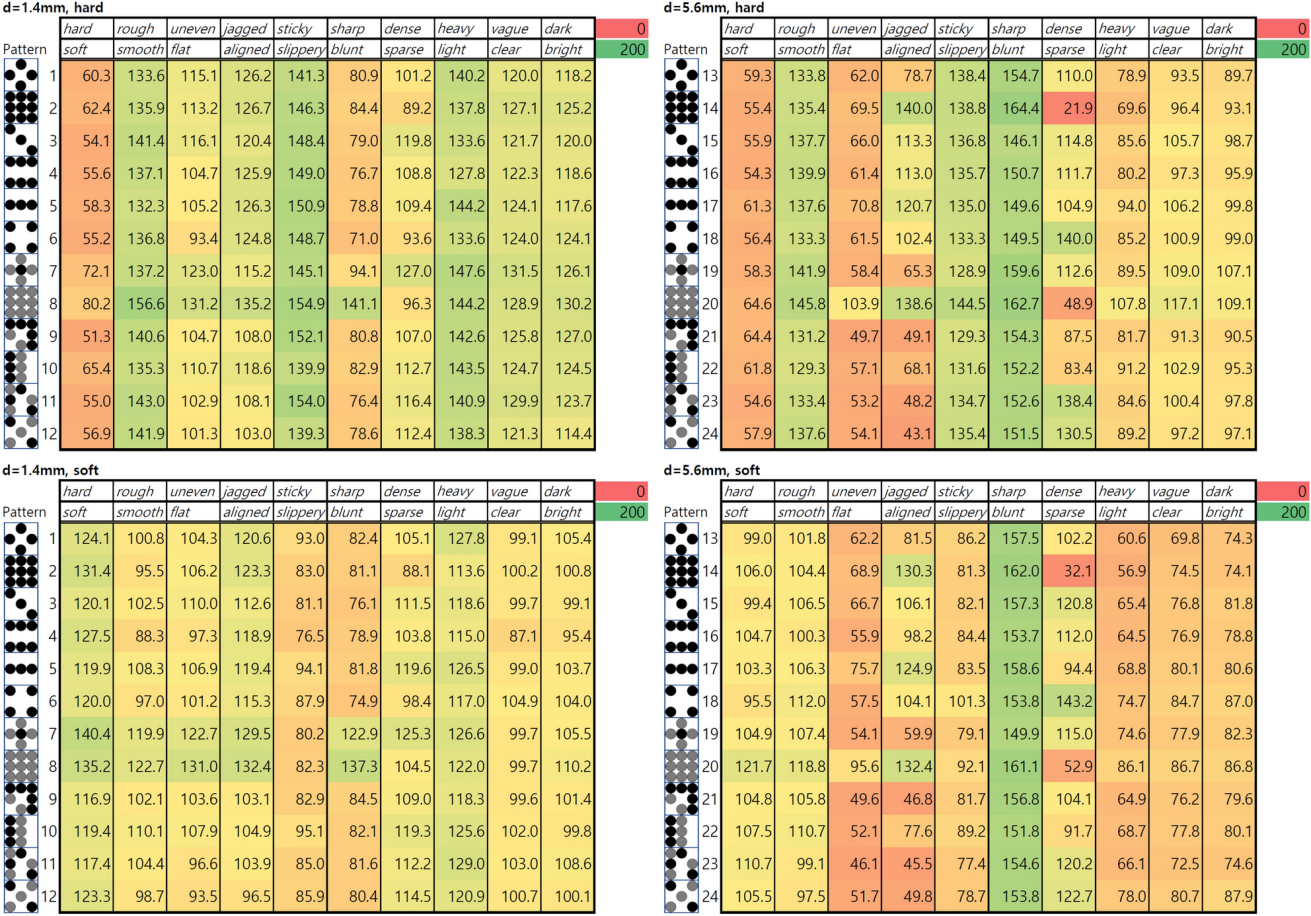
The color maps of average adjective ratings (0 to 200) by bump diameter, material, and patterns.

Figure 4 shows the color maps of the adjective rating results. Effects of the three main factors: material, bump diameter, and pattern, were analyzed in a three-way ANOVA test with repeated measures for the ten adjective pairs. As shown in Table 3, patterns had a significant effect on the subjective ratings for all adjective pairs. Meanwhile, materials and size affected the subject ratings for specific adjective pairs (stiffness, micro-roughness, macro-roughness, viscoelasticity, clarity, and brightness for material; macro-roughness, spatial regularity, sharpness, weight, clarity, and brightness for size). We observed no significant interaction between the factors for all adjective pairs. The color maps in Fig. 4 also indicate a trend that properties including ‘hard-soft’, ‘rough-smooth’, and ‘sticky-slippery’ are affected by the material of the samples ($p<0.001$). For example, soft samples, regardless of the size, tended to be felt stickier than the hard samples. Meanwhile, the ratings of the properties ‘uneven-flat’, ‘sharp-blunt’, ‘light-heavy’, and ‘dark-bright’ tended to be affected by the size of the samples ($p<0.01$). The smaller samples with a height of 1.4 mm tended to be felt flatter, sharper, and lighter than larger samples with a height of 5.6 mm.

Distributions of the participants’ ratings were diverse along the 13 kinds of patterns. In terms of average rating, participants reported the pattern samples were close to hard (hard-soft, avg. 87.3), smooth (rough-smooth, avg. 125.6), slippery (sticky-slippery, avg. 116.7), and blunt (sharp-blunt, avg. 123.3). They rated the flat pattern (no. 25) and equally low-height pattern (no. 8 and 20) as the most different patterns from the others. These patterns were regarded as the most smooth, flat, aligned, blunt, light, clear, and bright patterns.

Experimental results also show the effect of sample type on the perceived property intensities. When it comes to the samples with a height of 5.6 mm, samples with unevenly distributed patterns, such as 13, 19, 21, 22, 23, and 24, tended to feel more jagged than others. Evenly distributed patterns, 14 and 20, showed the trend of being felt denser than other samples. When the size of the samples is smaller, however, no noticeable effect of the sample type was observed.

**Fig. 5:**
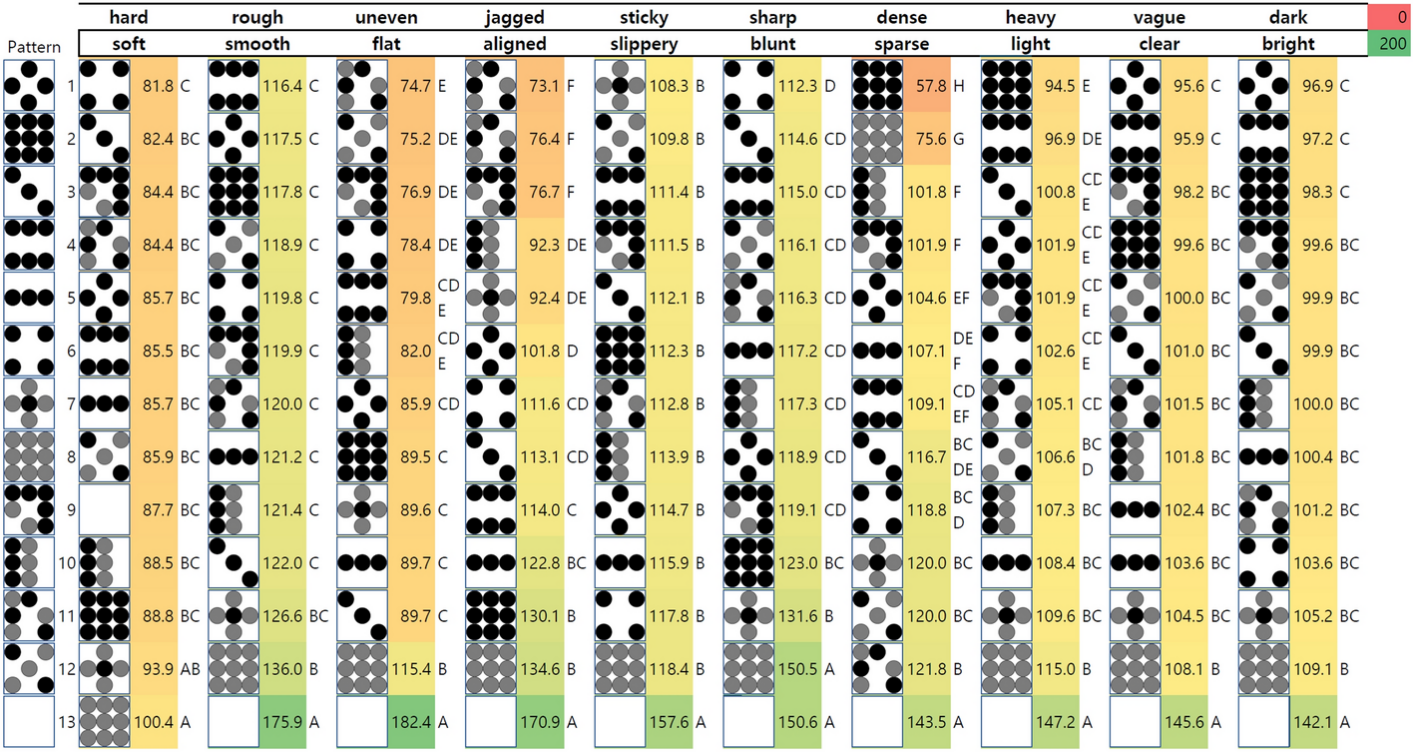
Sample patterns aligned by properties. Alphabets indicate sample subgroups for each property after Tukey’s HSD multiple comparison tests.

The distributions of ratings were also different along with adjective pairs. We conducted Tukey’s HSD multiple comparison tests to find statistically distinguished groups of patterns for ratings of each adjective pair. The number of statistical groups varied with adjectives and ranged from two to six. We represented each statistical group as an alphabet as shown in Fig. 5. The number of groups is different by the properties, which would correspond to the number of identifiable adjective stages that can be rendered with a 2.5D display.

**Fig. 6:**
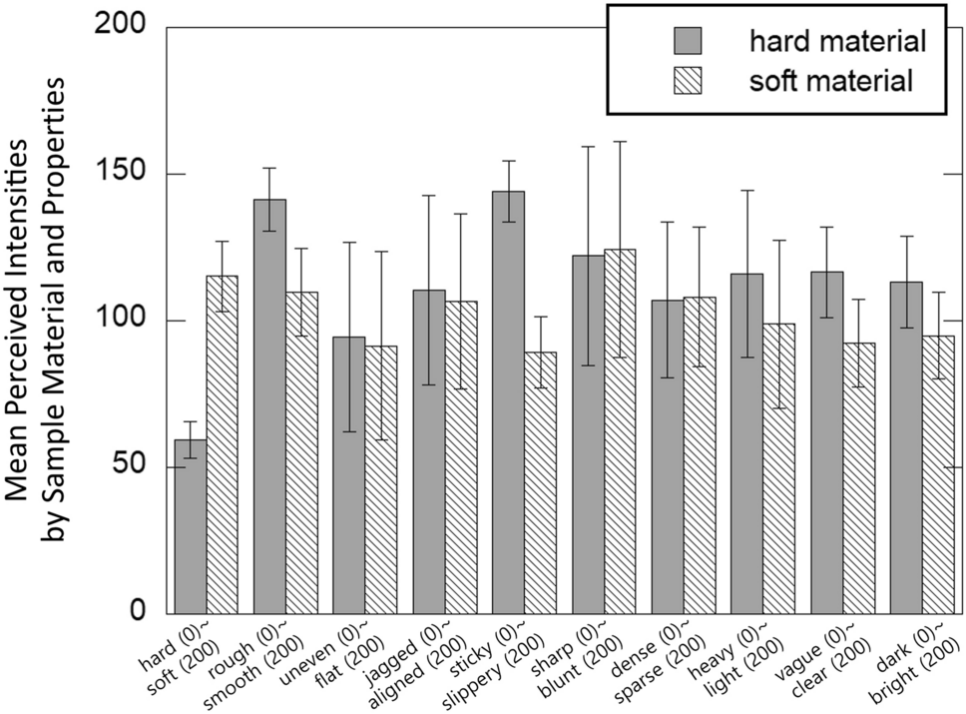
Mean perceived intensities by sample material and properties. Error bars indicate the standard deviations.

**Fig. 7:**
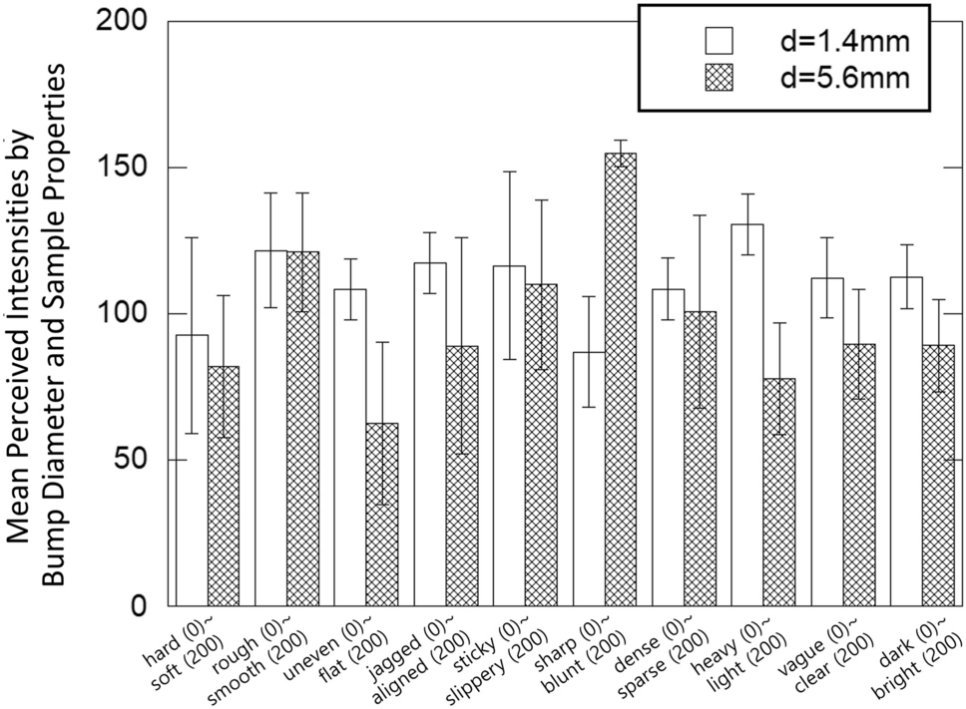
Mean perceived intensities by bump diameter and sample properties. Error bars indicate the standard deviations.

Figure 6 shows the mean perceived intensities by properties (adjective pairs) and sample material. As shown in Table 3, the effect of sample material is visible for specific properties, including stiffness, micro-roughness, macro-roughness, viscoelasticity, clarity, and brightness. Figure 7 shows the mean perceived intensities by properties and bump diameter. The effect of bump diameter is noticeable for all the properties except for micro-roughness, viscoelasticity, and spatial density.

**Table 3:** Data analysis results (*p*-values with *F*-scores in ANOVA test) of Experiment 1 by adjectives.

Adjective	Material	Bump diameter	Pattern
Stiffness	$p<$0.001, F(1,23) = 51.29	*p* = 0.111, F(1,23) = 2.87	$p<$0.001, F(11,253) = 7.86
Micro-roughness	$p<$0.001, F(1,23) = 28.18	*p* = 0.739, F(1,23) = 0.114	$p<$0.001, F(11,253) = 5.1
Macro-roughness	*p* = 0.019, F(1,23) = 6.35	$p<$0.001, F(1,23) = 37.25	$p<$0.001, F(11,253) = 15.63
Spatial regularity	*p* = 0.094, F(1,23) = 3.06	*p* = 0.001, F(1,23) = 14.29	$p<$0.001, F(11,253) = 35.76
Viscoelasticity	$p<$0.001, F(1,23) = 85.98	*p* = 0.42, F(1,23) = 4.67	*p* = 0.008, F(11,253) = 2.39
Sharpness	*p* = 0.094, F(1,23) = 3.05	$p<$0.001, F(1,23) = 161.73	$p<$0.001, F(11,253) = 27.96
Spatial density	*p* = 0.28, F(1,23) = 1.21	*p* = 0.7, F(1,23) = 0.154	$p<$0.001, F(11,253) = 23.62
Weight	*p* = 0.88, F(1,23) = 0.53	$p<$0.001, F(1,23) = 6.12	$p<$0.001, F(11,253) = 0.6
Clarity	$p<$0.001, F(1,23) = 22.61	*p* = 0.001, F(1,23) = 14.16	*p* = 0.044, F(11,253) = 1.87
Brightness	*p* = 0.001, F(1,23) = 14.94	*p* = 0.002 , F(1,23) = 12.63	*p* = 0.001, F(11,253) = 2.99

## Experiment 2: cluster sorting and perceptual space estimation of 2.5D tactile patterns

Experiment 2 aims to achieve a perceptual space of 2.5D tactile patterns formed by the adjective pairs used in Experiment 1. Thus, we conducted a cluster sorting task to measure the perceptual distances between each pair of 25 patterned 2.5D samples. Ward proposed the cluster-sorting for the MDS for the visual stimuli^33^. Later, MacLean’s group adopted the algorithm and experimental procedure to categorize the tactile stimuli group that is closely located in the perceptual space^24,29^. The 2.5D pattern samples were presented to a user by a custom-built experimental apparatus. A dissimilarity matrix was derived from the cluster sorting task, and the MDS was conducted to convert the measured perceptual distances into an Euclidean space for the perceived 2.5D tactile patterns. Consequently, the distribution and grouping of the 2.5D tactile patterns over the perceptual space formed by the adjective pairs are achieved from Experiment 2.

The experimental methods were approved by Hongik University Institutional Board with No. 7002340-202203-HR-002 and the experiment was performed in accordance with the Declaration of Helsinki.

### Methods

#### Participants

A total of twenty-four volunteers (seven male and seventeen female; 19–29 years old, mean±SD: 22.0±2.8) with no known sensory disorders participated in the experiment. Informed consent was obtained from all participants. All participants were right-handed by self-reporting and were compensated after participating in the experiment.

#### Stimuli

Twenty-five samples with hard surfaces described in Experiment 1 were used. Each sample was fabricated twice, making a total of fifty samples for Experiment 2.

#### Apparatus

**Fig. 8:**
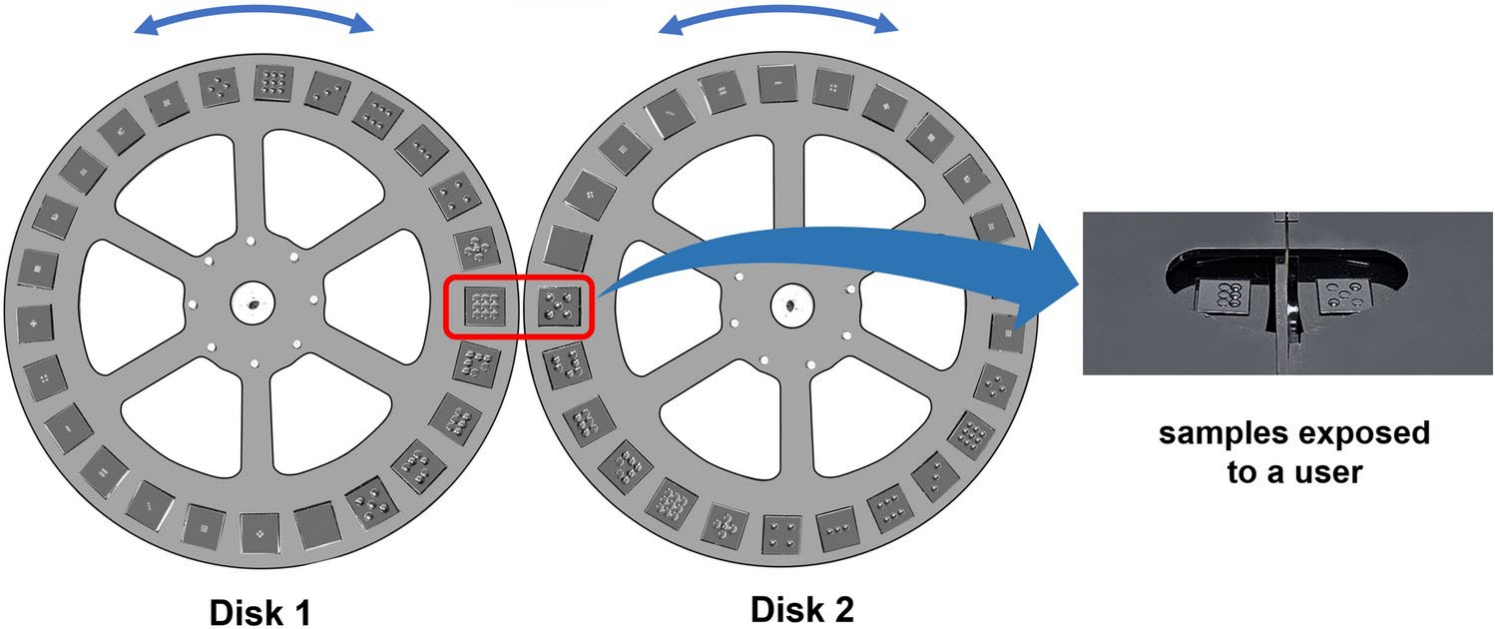
The experimental apparatus for Experiment 2 consists of two disks hidden inside two boxes. Twenty-five samples are installed on each disk, and two samples from each disk are exposed outside. During the experiment, a small box with a black cloth covers the samples so that a user cannot visually see the samples.

In a cluster sorting procedure, a participant should compare two different stimuli for grouping. Thus, we built a new experimental apparatus that can present a pair of 2.5D tactile patterns to a user, as requested by a computer program made with Unity (Fig. 8). The experimental apparatus consists of two boxes, each of which contains a rotating disk mechanism. A disk was made by stacking two laser-cut acrylic plates, each with a diameter of 29 cm and a thickness of 3 mm. On the disk, the twenty-five 2.5D samples described in the previous subsection are attached. Thus, when the rotating disk is installed inside the box, only one sample is exposed outside. Two boxes containing the disks are attached side-to-side, exposing a pair of samples to a user. A small box with a black cloth at the opening is installed over the samples, blocking visual cues from a user. The mechanism and the control method are the same as in Experiment 1.

#### Procedure

**Fig. 9:**
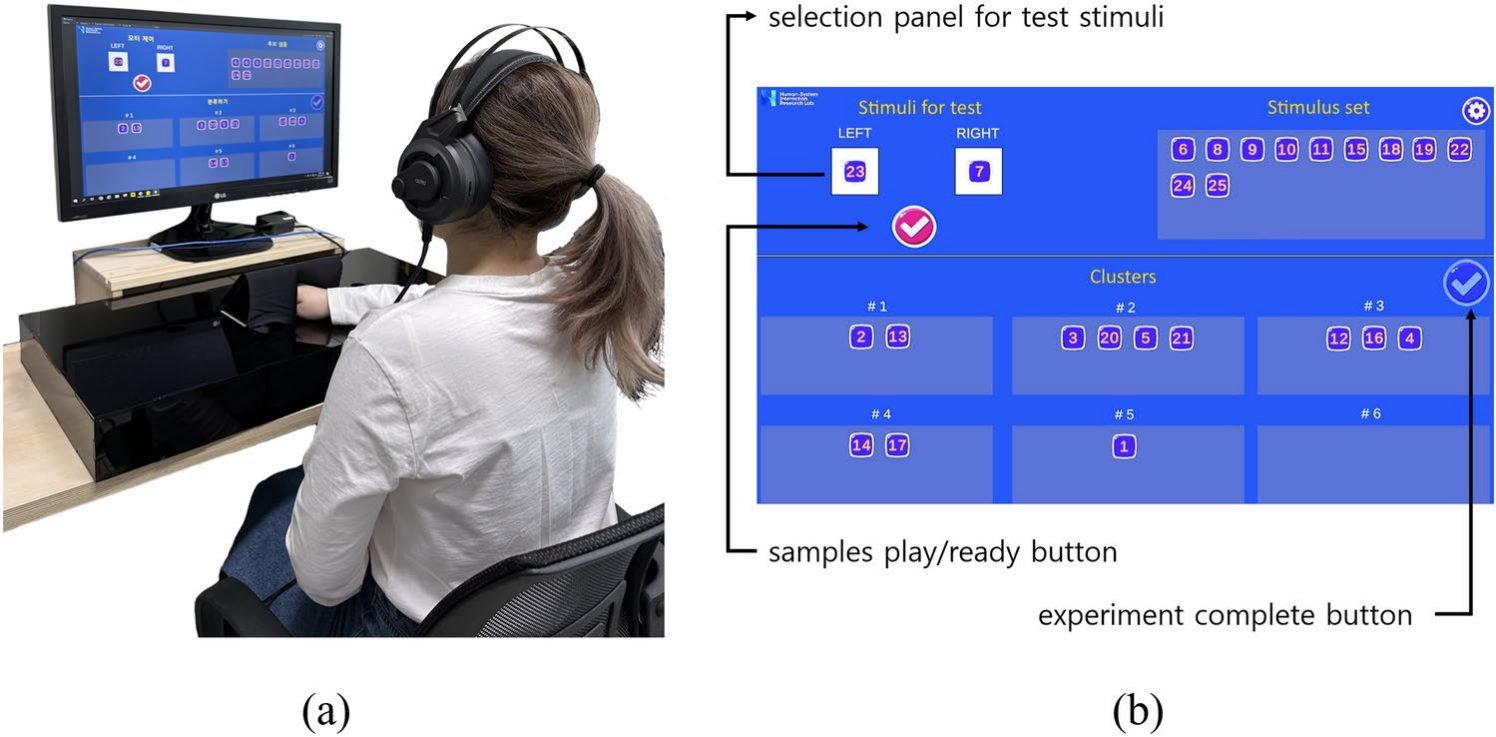
Setup for experiment 2 (**a**) A subject participating the experiment. (**b**) Experimental program.

At the beginning of the experiment, a participant was seated in front of an experimental computer and wore headphones where white noise was played during the experiment to block possible noise from the experimental apparatus (Fig. 9a). After the participant typed their ID with the keyboard, an instruction for the experiment appeared on the screen asking him/her to feel the samples and sort them into *N* clusters (*N* = 3, 6, 9). When clicking a ‘next’ button, the main experiment screen appeared as shown in Fig. 9b. The participant could drag a number from the stimulus set to either of blank ‘LEFT’ or ‘RIGHT’ square on the left. After two sample numbers were in the squares, the ‘play/ready’ button was activated with the sign ‘$\blacktriangleright$’. When the participant hit the button, the experimental apparatus moved the samples with the numbers on the program to the openings. As the experimental disk was moving, white noise was played to the headphones so that the participant could not hear the sound from the apparatus. When the desired samples were moved to the opening, ‘ready’ sign (’$\checkmark$’) appeared on the ‘play/ready’ button. Then, the participant felt the two samples with the fingers and judged how they were classified. After feeling the two samples, s/he dragged the numbers to the cluster boxes below on the experimental program. Once the participant judged that the cluster sorting was completed, they hit the ‘experiment complete’ button that ended the experiment. For the participant to get familiarized with the experimental procedure, the experiment for the first session was regarded as training, and thus, the data was discarded. When the experiment was complete, each participant’s results were stored both in the experimental computer and the local database for the data analysis. The cluster sorting was conducted for each of *N*, the number of clusters.

#### Data analysis

The data analysis was conducted by following a typical procedure for the cluster sorting^22,29^. Initially, the similarity score between the samples *i* and *j* $s_{i,j}$ was set to zero for each participant. When the samples *i* and *j* were grouped together in *N*-cluster test, *N* was added to the similarity score $s_{i,j}$. For example, if the samples numbered 3 and 4 were grouped together in 6 and 9-cluster tests, the similarity score $s_{3,4}$ would be increased by 3+6 = 9. By taking the average of $s_{i,j}$ for all the participants, we get the average similarity score $\bar{s}_{i,j}$ and the dissimilarity matrix $\left\{ d_{i,j} | 1\le i, j\le 25 \right\}$ as follows:

1 $$d_{{i,j}} = 1000\left( {1 - \frac{{\bar{s}_{{i,j}} }}{{3 + 6 + 9}}} \right),$$

where the constant 1000 is a scaling factor. Then, non-metric classical MDS was applied to the dissimilarity matrix to derive the Euclidean perceptual spaces. To see the classification of the texture points in the perceptual space, we used hierarchical clustering with the furthest neighbor metric^34^. We used the squared Euclidean distance for the interval measure. A dimensionality solution for the MDS was selected from a Kruskal’s stress plot.

Next, the adjective ratings from Experiment 1 were projected onto the MDS solution space by applying a multiple linear regression. For each adjective pair, standardized regression coefficients were derived with the stimulus coordinates as dependent variables and the ratings of the adjective pair as independent variables. The regression coefficients were treated as a vector in the MDS solution space for the adjective rating. Then, the adjective rating vector was scaled by the coefficient of determination, $R^2$, deciding the length of the vector displayed. It means that the longer an adjective pair line is, the higher the correlation between the adjective pair and the samples in the perceptual space.

**Fig. 10:**
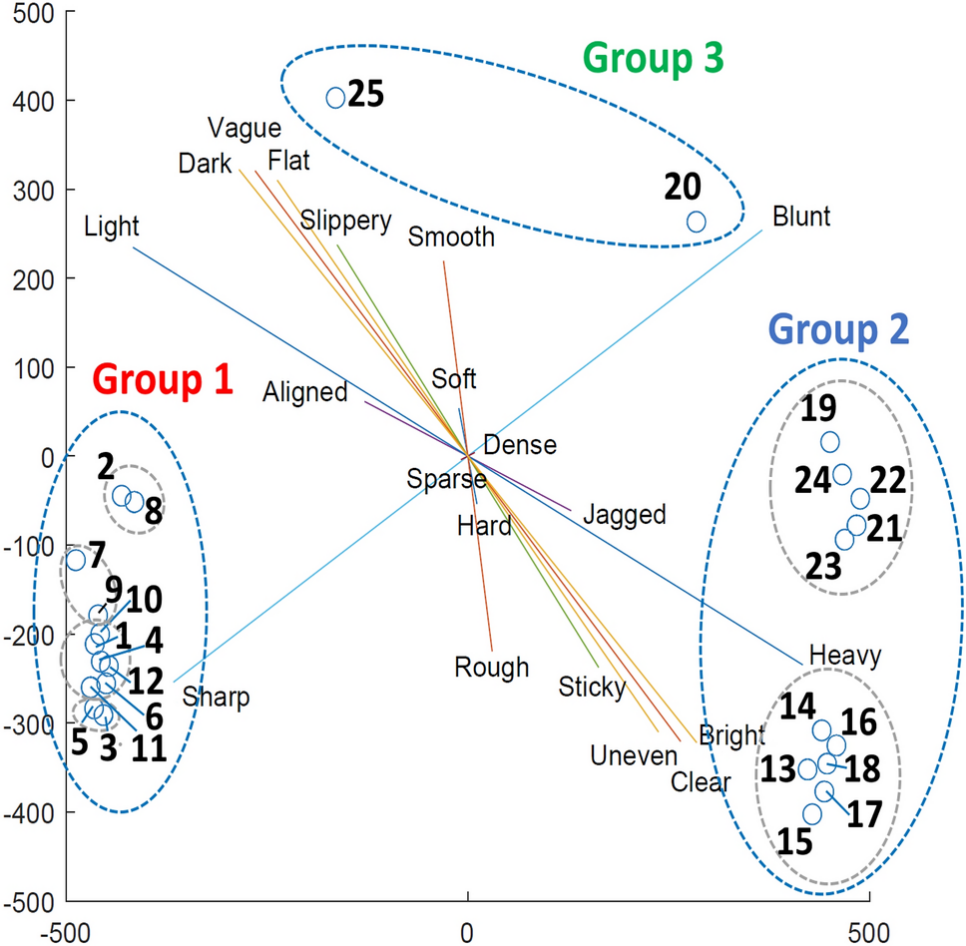
Adjective pairs regressed to the 2D perceptual space of the 2.5D tactile patterns.

**Fig. 11:**
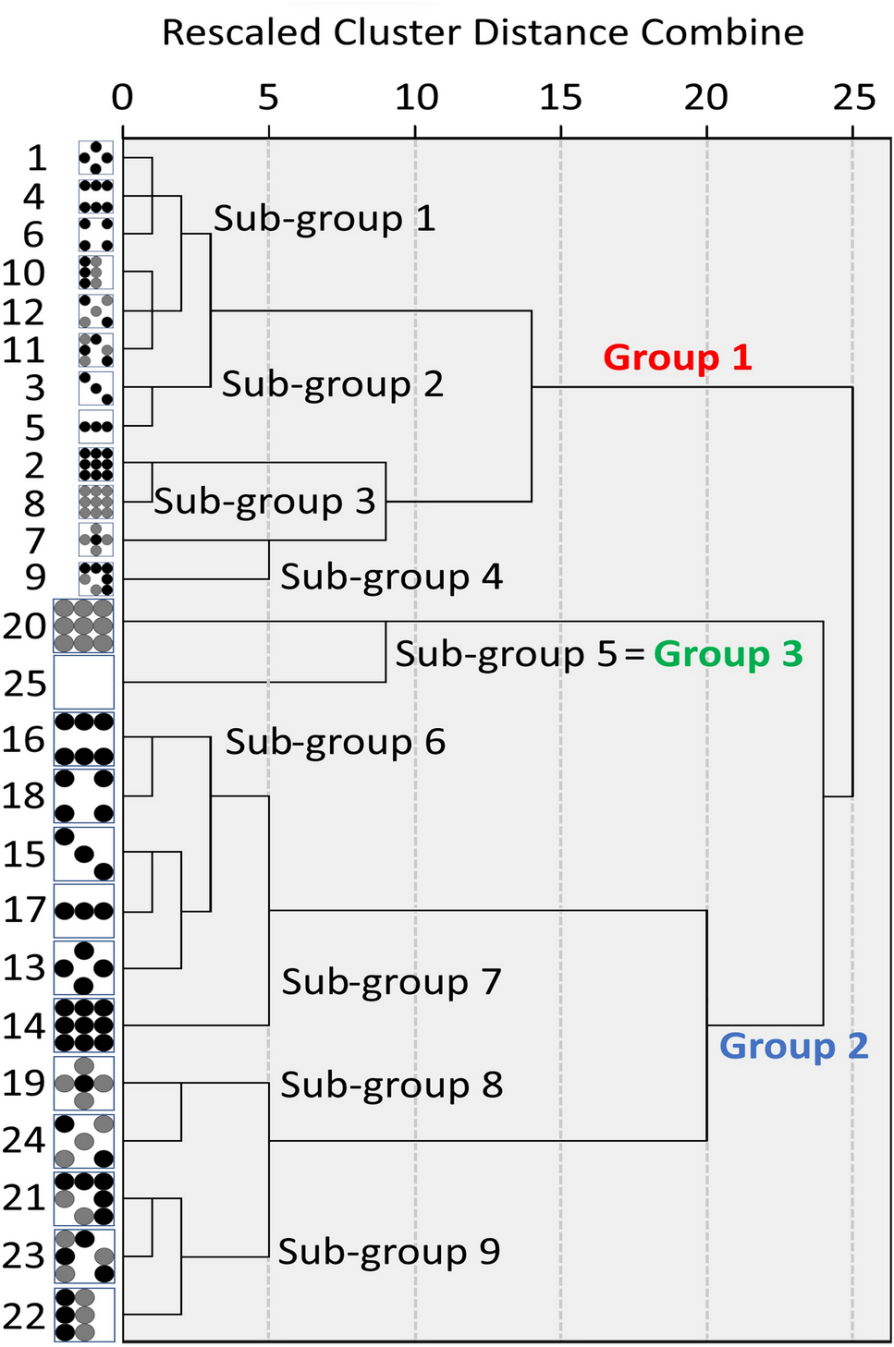
Dendrogram of the 2.5D tactile patterns’ hierarchical clustering.

### Results

With the dissimilarity matrix from the experimental result, we applied an MDS to derive a perceptual space for the distribution of the samples. The Kruskal’s Stress of the MDS saturated when there were two dimensions as 0.0542, indicating an ‘elbow point’ as a good fit of the data analysis^35^. The 2.5D tactile pattern points can be visualized in a 2D space as shown in Fig. 10. Figure 11 shows the dendrogram of the hierarchical clustering. Overall, the 2.5D tactile patterns can be classified into nine sub-groups which can be re-grouped into three groups.

Group 1 includes twelve samples {1, 2, 3, 4, 5, 6, 7, 8, 9, 10, 11, 12}, which is identical to the group with the size *d*  =  1.4 mm. Two samples, {20, 25}, are included in Group 2. Group 3 included eleven samples {13, 14, 15, 16, 17, 18, 19, 21, 22, 23, 24}, all of which have the size *d*  = 5.6 mm. Group 1 includes sub-groups 1, 2, 3, and 4, which consist of the samples {1, 4, 6}, {10, 11, 12}, {2, 8} and {7, 9}, respectively. Group 3 includes sub-group 6, 7, 8 and 9, which consist of the samples {13, 15, 16, 17, 18}, {14}, {19, 24} and {22, 23}. The samples of Groups 1 and 3 clearly show the size of the samples affected the participants’ clustering of the samples. Meanwhile, Group 2 indicates that the participants perceived a sample with low height and uniform distribution, i.e., sample 20, as being close to a flat surface, sample 25.

**Table 4:** Intersecting angles in degree between adjective axes.

	Rough-smooth	Uneven-flat	Simple-complex	Sticky-slippery	Sharp-blunt	Dense-sparse	Light-heavy	Clear-vague	Bright-dark
Hard-soft	$51.33^{\circ }$	$56.46^{\circ }$	$85.41^{\circ }$	$51.33^{\circ }$	$69.88^{\circ }$	$75.62^{\circ }$	$51.54^{\circ }$	$46.98^{\circ }$	$46.01^{\circ }$
Rough-smooth		$21.48^{\circ }$	$36.12^{\circ }$	$19.38^{\circ }$	$55.36^{\circ }$	$27.16^{\circ }$	$59.91^{\circ }$	$28.22^{\circ }$	$31.63^{\circ }$
Uneven-flat			$34.4^{\circ }$	$9.48^{\circ }$	$76.83^{\circ }$	$42.57^{\circ }$	$40.97^{\circ }$	$10.1^{\circ }$	$13.58^{\circ }$
Simple-complex				$25.29^{\circ }$	$73.74^{\circ }$	$28.31^{\circ }$	$68.4^{\circ }$	$43.91^{\circ }$	$46.85^{\circ }$
Sticky-slippery					$73.22^{\circ }$	$34.68^{\circ }$	$49.23^{\circ }$	$19.53^{\circ }$	$22.91^{\circ }$
Sharp-blunt						$45.47^{\circ }$	$69.28^{\circ }$	$82.24^{\circ }$	$85.13^{\circ }$
Dense-sparse							$83.53^{\circ }$	$127.99^{\circ }$	$124.36^{\circ }$
Light-heavy								$31.96^{\circ }$	$28.4^{\circ }$
Clear-vague									$3.63^{\circ }$

**Fig. 12:**
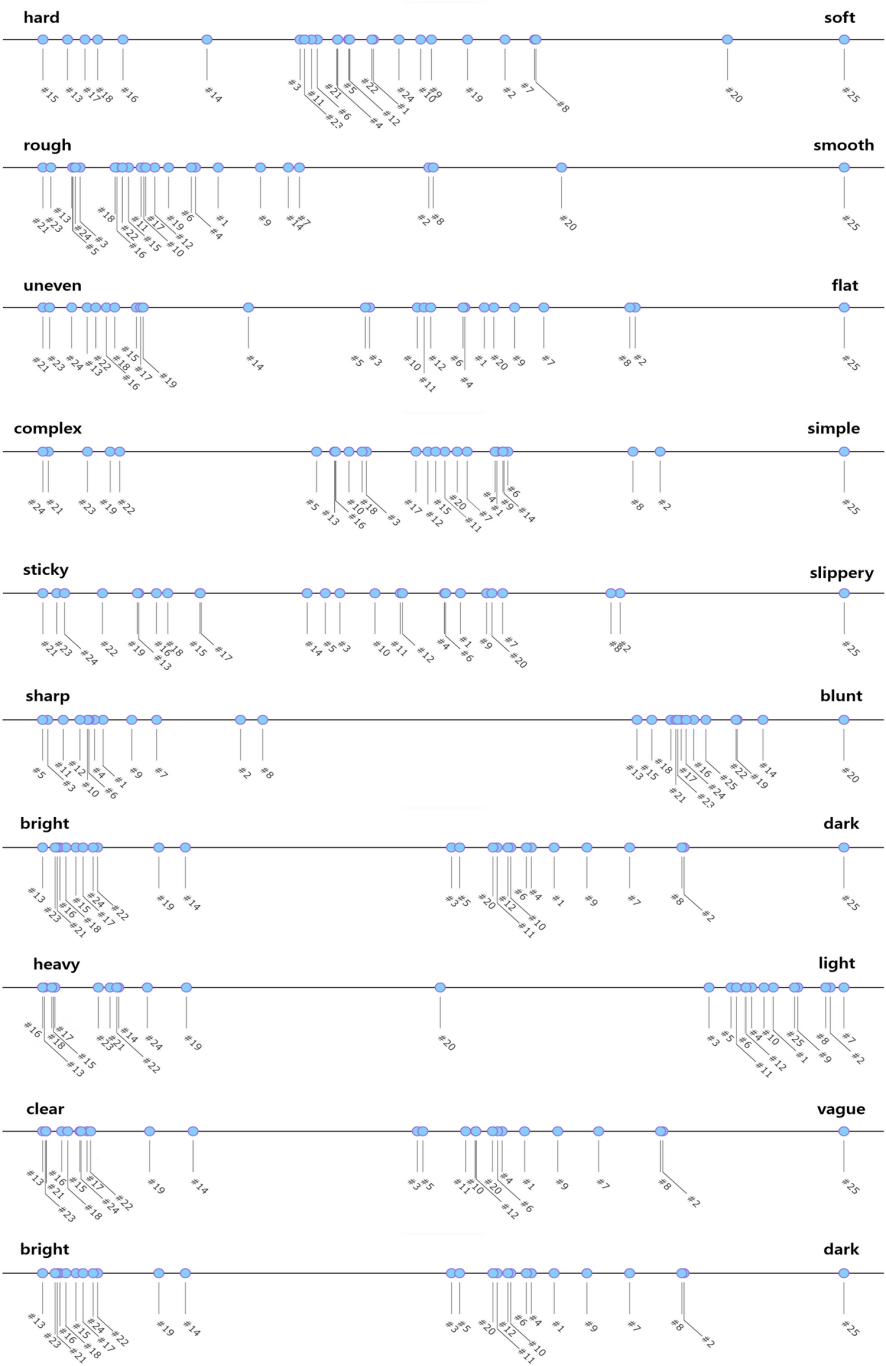
Projection of the 2.5D tactile patterns with hard surface onto adjective pair lines.

Figure 10 shows the adjective pairs represented as lines intercepting the origin with a slope proportional to the ratio of standardized regression coefficients along with sample points. Table 4 shows the angular difference between adjective axes. From Fig. 10, four adjective pairs, i.e., heavy-light, dark-bright, vague-clear, and flat-uneven, are prominently correlated to the 2.5D samples. Six samples, 13, 14, 15, 16, 17, and 18, show a high correlation to the adjective pairs, which commonly have larger bump size (*d*  =  5.6 mm) and high average bump height (*h*  =  1.44 mm). It is also observed that eight samples, 1, 3, 4, 5, 9, 10, 11, 12, and 20 are highly correlated to the adjective pair, sharp-blunt. From Fig. 10 and Table 4, the angle between the previous four adjective pairs and the sharp-blunt pair is close to 90 degrees, showing the orthogonality of the perceptual dimensions. Except for sample 20, which is biased to the blunt side, all other samples have a smaller size (*d*  =  1.4 mm) and are biased toward the sharp side. Figure 12 shows how 2.5D samples are projected to line segments for adjective pairs. The distributions of 2.5D samples on the adjective axes show localized polarities, coinciding with the results in Table 3 and Fig. 10.

## Discussions

The present study investigated the human perception of 2.5D tactile patterns in the context of the perceptual space based on adjectives and how physical parameters such as material and the pattern’s bump diameter affected the haptic perception. From the results of Experiment 1, a significant effect of the types of 2.5D patterns was observed for the perceived intensities for adjective pairs. The experimental results indicate that the material and size of the samples affected the perception of the 2.5D samples’ properties. From the result of cluster sorting in Experiment 2, the 2.5D samples were projected to 2D perceptual spaces with the MDS. The clustering shows a clear trend of grouping by the size of the 2.5D samples. It was also observed that the 2.5D samples were grouped by the randomness or uniformity when the bump diameter was large (*d*  =  5.6 mm).

A notable feature of Experiment 1 is that the sample materials affected the perceived intensities of specific properties. The clear trend of sample material affecting the perceived intensity of the stiffness can be readily explained, noting the two materials used for the experiment differed in stiffness. The significant effect of sample material on the perceived viscoelasticity can be ascribed to the difference in the friction between the soft and hard material. Previous studies indicate that the dominant factor in the perceived stickiness/friction is the material’s adhesion, which is higher on the soft surface than the hard surface^36,37^. For Experiment 1, the surface of soft samples would have increased the friction between the bare fingertip and the surface, leading to an increased intensity of the stickiness. The effect of the sample material on the micro-roughness (‘rough-smooth’) can be ascribed to the increase or decreased exploratory speed by the type of the material. The major factor affecting the perceived roughness of texture can be thought of as the surface element’s density^38^. However, the effect of velocity was also found to affect the perception of roughness, which would increase on a hard sample surface than a soft one fabricated with silicone.

Experiment 2 resulted in the grouping of the 2.5D tactile patterns, which showed a clear trend for the samples with *d*  =  5.6 mm, which is the grouping by the uniformity/randomness of the patterns. As Fig. 11 shows, the large samples were clearly divided into two subgroups by the randomness of the spatial and height distribution, say, uniform patterns (samples numbered between 14 and 19) and random patterns (samples numbered 19, 21, 22, 23, and 24). Considering the larger samples’ size, they can be classified as macrotextures, which also coincides with the result of Experiment 1, where bump diameter affected the perceived intensity of the property ‘macro-roughness’^37^. This means that the larger samples are neurally processed as spatial arrays, whose information is mainly delivered by slowly adapting mechanoreceptors^39,40^. Thus, the participants are thought to have perceived the large patterns as spatial arrays and perceived the random and uniform patterns differently with different deformation patterns at the fingertip. An alternative explanation can be found in the Weber-Fechner law. The height difference for the random patterns would have been over the JND of a bump height perception. Then, additional height difference information would have been provided to a participant, resulting in distinctive grouping by the height variation.

Another notable feature of Experiment 2 is that the bump diameter significantly affected their grouping. As shown in Fig. 11, the smaller samples (*d* = 1.4 mm) and larger samples (*d* = 5.6 mm) were classified in two different groups, Group 1 and 2, respectively. An explanation can be found in the different neural processes as the size of the texture varied. An exploration over a fine texture creates vibratory signals stimulating Pacinian corpuscles, while a coarse texture exploration results in the stimulation of slowly adapting mechanoreceptors^41,42^. A significant difference in bump diameter between the two groups would have caused different neural processing of input signals from different combinations of mechanoreceptor channels. It also explains the result of Experiment 1, where the perceived intensities of specific properties showed opposite trends by the bump diameter. A similar trend is found in Hwang and Choi’s previous work^26^, where vibrotactile signals were grouped distinctively as the signal’s amplitude changed. As is the case of the current study, variation of vibrotactile signal would have stimulated mechanoreceptor in a different manner, considering that the Pacinian corpuscles are located in a lower layer down to the skin, being sensitive to the signal intensity. Therefore, the distinctive groupings of Experiment 2 by the bump diameter can be explained by the difference in the neural process, i.e., the stimulation of Pacinian corpuscles when exploring over smaller samples. Similarly, the distinctive subgroups for the larger patterns could have resulted from the difference in stimulating the slowly adapting mechanoreceptors.

The perceptual space derived from the current study can be compared to the previous studies that resulted in the perceptual space of texture from real physical objects, e.g., the study by Hollins et al.^23^. In the previous study, it is notable that the dimension for ‘soft-hard’ is perpendicular to other properties, such as ‘smooth-rough’ or ‘slippery-sticky.’ In the current study, as shown in Fig. 10 and Table 4, the angular difference from ‘soft-hard’ to other properties vary between 45 and 85 degrees, not as perpendicular as was for the Hollins et al.’s study. The difference can be partially explained by the difference in the variation of the tactile specimen, where the current experiment used samples of a specific size, dimension, and materials. In contrast, the previous study used various physical objects. Moreover, as discussed in the previous paragraph, the control of sample properties, such as texture size or material, in the current study resulted in a distinctive polarity, which can significantly affect the dimension of perceptual space. When compared to Hwang et al.’s study, which analyzed the perceptual space of vibrotactile signals^26^, the correlation between properties shows a different trend than the current study. As was for the comparison with Hollins et al.’s study, the discrepancy can be ascribed to the difference in the channels to process the tactile stimuli.

To summarize, the present study quantitatively analyzed the human perception of 2.5D tactile patterns by constructing the perceptual space and investigating the effect of parameters on the perceived properties, including the samples’ material, bump diameter, and randomness. Significant effects of the parameters on the perceived intensities and grouping were observed from the experimental results, which will serve as a ground for designing an effective surface-morphing tactile display. For example, the grouping of samples with *d* = 5.6 mm implies that the surface-morphing display can render a broader range of tactile sensations if it can effectively change the surface height when the tactile resolution is relatively low. In our future work, we plan to investigate further the human perception of 2.5D tactile patterns with more extensive stimuli, including the tactile patterns rendered by the surface-morphing display.

## Data Availability

The datasets used and/or analysed during the current study are available from the corresponding author on reasonable request.

## References

[1] Hwang, I. et al. Height-renderable morphable tactile display enabled by programmable modulation of local stiffness in photothermally active polymer. *Nat. Commun.* **15**, 2554 (2024). 38519461 10.1038/s41467-024-46709-7 PMC10959967

[2] Besse, N. et al. Understanding graphics on a scalable latching assistive haptic display using a shape memory polymer membrane. *IEEE Trans. Haptics* **11**, 30–38 (2018). 29611811 10.1109/TOH.2017.2767049

[3] Besse, N., Rosset, S., Zarate, J. J. & Shea, H. Flexible active skin: Large reconfigurable arrays of individually addressed shape memory polymer actuators. *Adv. Mater. Technol.* **2**, 1700102 (2017).

[4] Qiu, Y., Lu, Z. & Pei, Q. Refreshable tactile display based on a bistable electroactive polymer and a stretchable serpentine Joule heating electrode. *ACS Appl. Mater. Interfaces* **10**, 24807–24815 (2018). 29968468 10.1021/acsami.8b07020

[5] Zhang, K., Gonzalez, E. J., Guo, J. & Follmer, S. Design and analysis of high-resolution electrostatic adhesive brakes towards static refreshable 2.5 D tactile shape display. *IEEE Trans. Hapt.* **12**, 470–482 (2019). 10.1109/TOH.2019.2940219 31545743

[6] Kreimeier, J., Karg, P. & Götzelmann, T. Tabletop virtual haptics: Feasibility study for the exploration of 2.5 D virtual objects by blind and visually impaired with consumer data gloves. In *Proceedings of the 13th ACM International Conference on Pervasive Technologies Related to Assistive Environments*, 1–10 (2020).

[7] Kim, G., Hwang, D. & Park, J. Effect of 2.5 D haptic feedback on virtual object perception via a stylus. *Sci. Rep.* **11**, 18954 (2021). 34556780 10.1038/s41598-021-98589-2 PMC8460700

[8] Kim, Y., Kim, D. & Ryu, J.-H. Haptic field and force feedback generation for wheeled vehicle teleoperation on 2.5 D environments. In *International Conference on Intelligent Autonomous Systems*, 85–94 (Springer, 2023).

[9] Park, C., Hong, S. & Park, J. Effect of rendering virtual vibrotactile motion on the perceived lateral force. *IEEE Access* **12**, 173792–173799 (2024).

[10] Russomanno, A., O’Modhrain, S., Gillespie, R. B. & Rodger, M. W. Refreshing refreshable braille displays. *IEEE Trans. Haptics* **8**, 287–297 (2015). 25879973 10.1109/TOH.2015.2423492

[11] Bolanowski, S. J. Jr., Gescheider, G. A., Verrillo, R. T. & Checkosky, C. M. Four channels mediate the mechanical aspects of touch. *J. Acoust. Soc. Am.* **84**, 1680–1694 (1988). 3209773 10.1121/1.397184

[12] Gescheider, G. A., Wright, J. H. & Verrillo, R. T. *Information-Processing Channels in the Tactile Sensory System: A Psychophysical and Physiological Analysis* (Psychology press, 2010).

[13] Gescheider, G. A. & Wright, J. H. Roughness perception in tactile channels: Evidence for an opponent process in the sense of touch. *Somatosens. Motor Res.* **30**, 120–132 (2013). 10.3109/08990220.2013.779242 23952287

[14] Johnson, K. O. The roles and functions of cutaneous mechanoreceptors. *Curr. Opin. Neurobiol.* **11**, 455–461 (2001). 11502392 10.1016/s0959-4388(00)00234-8

[15] Johnson, K. O. & Phillips, J. R. Tactile spatial resolution. I. Two-point discrimination, gap detection, grating resolution, and letter recognition. *J. Neurophysiol.* **46**, 1177–1192 (1981). 7320742 10.1152/jn.1981.46.6.1177

[16] Van Boven, R. W. & Johnson, K. O. The limit of tactile spatial resolution in humans: Grating orientation discrimination at the lip, tongue, and finger. *Neurology* **44**, 2361–2361 (1994). 7991127 10.1212/wnl.44.12.2361

[17] Goodwin, A., Browning, A. & Wheat, H. Representation of curved surfaces in responses of mechanoreceptive afferent fibers innervating the monkey’s fingerpad. *J. Neurosci.* **15**, 798–810 (1995). 7823181 10.1523/JNEUROSCI.15-01-00798.1995 PMC6578298

[18] LaMotte, R. H. & Srinivasan, M. A. Neural encoding of shape: Responses of cutaneous mechanoreceptors to a wavy surface stroked across the monkey fingerpad. *J. Neurophysiol.* **76**, 3787–3797 (1996). 8985876 10.1152/jn.1996.76.6.3787

[19] Lawrence, M. A., Kitada, R., Klatzky, R. L. & Lederman, S. J. Haptic roughness perception of linear gratings via bare finger or rigid probe. *Perception* **36**, 547–557 (2007). 17564201 10.1068/p5746

[20] Kitada, R., Sadato, N. & Lederman, S. J. Tactile perception of nonpainful unpleasantness in relation to perceived roughness: Effects of inter-element spacing and speed of relative motion of rigid 2-d raised-dot patterns at two body loci. *Perception* **41**, 204–220 (2012). 22670348 10.1068/p7168

[21] Bernard, C., Monnoyer, J., Ystad, S. & Wiertlewski, M. Eyes-off your fingers: Gradual surface haptic feedback improves eyes-free touchscreen interaction. In *Proceedings of the 2022 CHI Conference on Human Factors in Computing Systems*, 1–10 (2022).

[22] Mun, S., Lee, H. & Choi, S. Perceptual space of regular homogeneous haptic textures rendered using electrovibration. In *2019 IEEE World Haptics Conference (WHC)*, 7–12 (IEEE, 2019).

[23] Holliins, M., Faldowski, R., Rao, S. & Young, F. Perceptual dimensions of tactile surface texture: A multidimensional scaling analysis. *Percept. Psychophys.* **54**, 697–705 (1993). 8134240 10.3758/bf03211795

[24] MacLean, K. & Enriquez, M. Perceptual design of haptic icons. In *Proc. of EuroHaptics*, 351–363 (2003).

[25] Ternes, D. & MacLean, K. E. Designing large sets of haptic icons with rhythm. In *Haptics: Perception, Devices and Scenarios: 6th International Conference, EuroHaptics 2008 Madrid, Spain, June 10-13, 2008 Proceedings 6*, 199–208 (Springer, 2008).

[26] Hwang, I. & Choi, S. Perceptual space and adjective rating of sinusoidal vibrations perceived via mobile device. In *2010 IEEE Haptics Symposium*, 1–8 (IEEE, 2010).

[27] Hwang, I., Seo, J. & Choi, S. Perceptual space of superimposed dual-frequency vibrations in the hands. *PLoS ONE* **12**, e0169570 (2017). 28081187 10.1371/journal.pone.0169570 PMC5230860

[28] Gescheider, G. A. & Wright, J. H. Perception of the tactile texture of raised-dot patterns: Further evidence of an opponent process in the sense of touch. *Somatosens. Motor Res.* **35**, 59–68 (2018). 10.1080/08990220.2018.1460262 29706104

[29] Pasquero, J., Luk, J., Little, S. & MacLean, K. Perceptual analysis of haptic icons: An investigation into the validity of cluster sorted MDS. In *2006 14th Symposium on Haptic Interfaces for Virtual Environment and Teleoperator Systems*, 437–444 (IEEE, 2006).

[30] Hollins, M., Bensmaïa, S., Karlof, K. & Young, F. Individual differences in perceptual space for tactile textures: Evidence from multidimensional scaling. *Percept. Psychophys.* **62**, 1534–1544 (2000). 11140177 10.3758/bf03212154

[31] Nagano, H., Okamoto, S. & Yamada, Y. Visual and sensory properties of textures that appeal to human touch. *Int. J. Affect. Eng.* **12**, 375–384 (2013).

[32] Okamoto, S., Nagano, H. & Yamada, Y. Psychophysical dimensions of tactile perception of textures. *IEEE Trans. Haptics* **6**, 81–93 (2012). 10.1109/TOH.2012.32 24808270

[33] Ward, L. M. Multidimensional scaling of the molar physical environment. *Multivar. Behav. Res.* **12**, 23–42 (1977). 10.1207/s15327906mbr1201_2 26804142

[34] Boberg, J. & Salakoski, T. General formulation and evaluation of agglomerative clustering methods with metric and non-metric distances. *Pattern Recogn.* **26**, 1395–1406 (1993).

[35] Dugard, P., Todman, J. & Staines, H. *Approaching Multivariate Analysis: A Practical Introduction* (Taylor & Francis, 2022).

[36] Adams, M. J., Briscoe, B. J. & Johnson, S. A. Friction and lubrication of human skin. *Tribol. Lett.* **26**, 239–253 (2007).

[37] Klatzky, R. L., Pawluk, D. & Peer, A. Haptic perception of material properties and implications for applications. *Proc. IEEE* **101**, 2081–2092 (2013).

[38] Lederman, S. J. Tactile roughness of grooved surfaces: The touching process and effects of macro-and microsurface structure. *Percept. Psychophys.* **16**, 385–395 (1974).

[39] Johnson, K. O., Hsiao, S. S. & Yoshioka, T. Neural coding and the basic law of psychophysics. *Neuroscientist* **8**, 111–121 (2002). 11954556 10.1177/107385840200800207 PMC1994651

[40] Blake, D. T., Hsiao, S. S. & Johnson, K. O. Neural coding mechanisms in tactile pattern recognition: The relative contributions of slowly and rapidly adapting mechanoreceptors to perceived roughness. *J. Neurosci.* **17**, 7480–7489 (1997). 9295394 10.1523/JNEUROSCI.17-19-07480.1997 PMC6573449

[41] Bensmaïa, S. & Hollins, M. Pacinian representations of fine surface texture. *Percept. Psychophys.* **67**, 842–854 (2005). 16334056 10.3758/bf03193537

[42] Bensmaïa, S., Hollins, M. & Yau, J. Vibrotactile intensity and frequency information in the Pacinian system: A psychophysical model. *Percept. Psychophys.* **67**, 828–841 (2005). 16334055 10.3758/bf03193536

